# Mitochondrial heteroplasmy in an avian hybrid form (*Passer italiae*: Aves, Passeriformes)

**DOI:** 10.1080/23802359.2019.1682477

**Published:** 2019-10-26

**Authors:** Martin Päckert, Gabriele Giacalone, Mario Lo Valvo, Christian Kehlmaier

**Affiliations:** a Senckenberg Naturhistorische Sammlungen, Dresden, Germany;; b Cooperativa Silene, Palermo, Italy;; c Dipartimento di Scienze e Tecnologie Biologiche Chimiche e Farmaceutiche, Universita degli Studi di Palermo, Palermo, Italy

**Keywords:** Paternal leakage, hybridization, sparrows, Mediterranean

## Abstract

Mitochondrial heteroplasmy is the result from biparental transmission of mitochondrial DNA (mtDNA) to the offspring. In such rare cases, maternal and paternal mtDNA is present in the same individual. Though recent studies suggested that mtDNA heteroplasmy might be more common than previously anticipated, that phenomenon is still poorly documented and was mostly detected in case studies on hybrid populations. The Italian sparrow, *Passer italiae* is a homoploid hybrid form that occurs all across the Italian Peninsula mostly under strict absence of either of its parent species, the house sparrow (*P. domesticus*) and the Spanish sparrow (*P. hispaniolensis*). In this study, we document a new case of mitochondrial heteroplasmy from two island populations of *P. italiae* (Ustica and Lipari). Our analysis was based on the mitochondrial NADH dehydrogenase subunit 2 (ND2) that allows for a clear distinction between mitochondrial lineages of the two parental species. We amplified and sequenced the mitochondrial ND2 gene with specifically designed primer combinations for each of the two parental species. In two of our study populations, a single individual carried two different ND2 haplotypes from each of the two parental lineages. These findings contribute to current knowledge on the still poorly documented phenomenon of paternal leakage in vertebrates.

## Introduction

In animals, strict maternal inheritance of mitochondrial DNA (mtDNA) is considered the rule, whereas bipaternal inheritance of mtDNA is regarded as an atypical and rather occasional exception (review in Breton and Stewart 2015). The latter rare phenomenon is known under the term ‘paternal leakage’, i.e. the transmission of male parent’s mitochondria to the ovum and thus to the heteroplasmic offspring. As the basic result of paternal leakage, mtDNA heteroplasmy defines the state when both paternal and maternal mtDNA are present in one individual (Breton and Stewart 2015; Mastrantonio et al. 2019). To date, there are only a few case reports of mitochondrial heteroplasmy in a few species all across the animal kingdom. Most of them refer to interspecific crosses that were occasionally documented as a byproduct from hybrid zone studies (as discussed in Bromham et al. 2002; Gandolfi et al. 2017). In some invertebrates, paternal leakage appears to be more frequent due to specific modes of fertilization (Meusel and Moritz 1993; Nunes et al. 2013; Wolff et al. 2013; Dokianakis and Ladoukakis 2014; Meza-Lázaro et al. 2018). However, a recent paper listed only 24 known case studies of paternal leakage in arthropods (ten of these within or among *Drosophila* species) including a new case study from *Rhipicephalus* ticks (Mastrantonio et al. 2019). However, many of the documented cases refer to experimental crosses (e.g. Adineh and Ross 2019), whereas examples from populations in the wild still appear to be rare. In some of the few examples from vertebrates, mtDNA heteroplasmy was suggested to be correlated with size polymorphism of mtDNA (Bermingham et al. 1986; Wolff et al. 2008). The latter examples of length heteroplasmy are generally distinguished from cases of point heteroplasmy, where maternal and paternal haplotypes of heteroplasmic individuals differ at single nucleotide positions (Just et al. 2015).

In this study, we document a further case of point heteroplasmy in a passerine bird. Examples from birds are also rare and refer to case studies of hybrid zones (Kvist et al. 2003) and to a particular stabilized hybrid form, the Italian sparrow, *Passer italiae* (Elgvin et al. 2017; Runemark et al. 2018). This homoploid hybrid form (Schumer et al. 2014) emerged from past hybridization between the house sparrow, *P. domesticus*, and the Spanish sparrow, *P. hispaniolensis* (Elgvin et al. 2011, 2017; Hermansen et al. 2011, 2014). It occurs all across the Italian Peninsula and on several Mediterranean islands mostly under the strict absence of either of its parent species (del Hoyo and Collar 2016). Italian sparrows (*P. italiae*) almost exclusively carry the mtDNA lineage of the house sparrow, whereas the Spanish sparrow mtDNA lineage is almost absent in Italian populations (Hermansen et al. 2011). In North Africa, a mosaic hybrid zone extends from eastern Morocco to Tunisia, where phenotypical hybrid populations occur in close vicinity with both parental species (Belkacem et al. 2016). In the following, we briefly report on a case of heteroplasmic individuals of *P. italiae* from two Mediterranean island populations.

## Materials and methods

**Figure 1.**
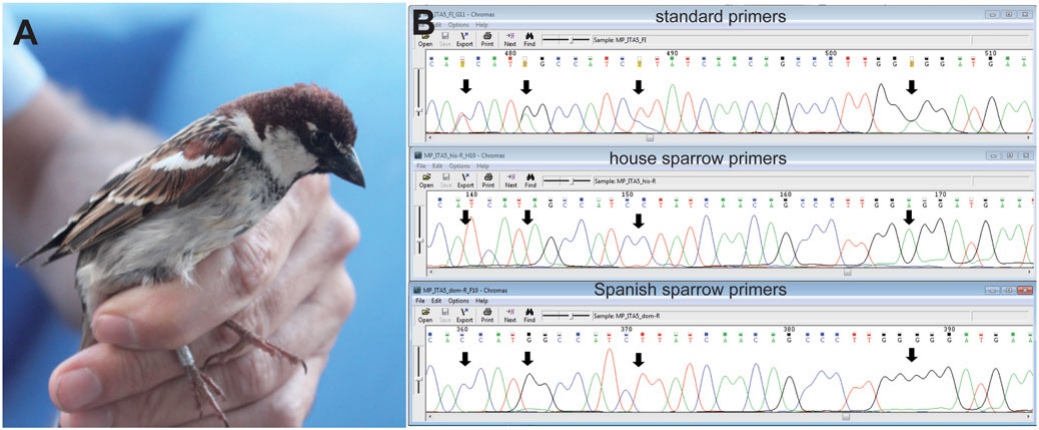
MtDNA heteroplasmy in one Italian sparrow, *P. italiae*; (A) ITA5 from Ustica island; photo M. Lo Valvo; (B) chromatograms of ND2 sequences, from three independent PCRs and sequencing reactions with standard primers and specific primers for mtDNA lineages of the house sparrow, *P. domesticus*, and the Spanish sparrow, *P. hispaniolensis*; segregating sites indicated by bold arrows.

In a previous study on mitochondrial introgression among North African sparrow hybrids and the two parental species we had amplified and sequenced NADH dehydrogenase subunit 2 (ND2) as a marker gene (Belkacem et al. 2016). In our sampling used for that study, the sequences inferred from two individuals of *P. italiae* from Mediterranean islands (ITA5 from Ustica (Figure 1(A)) and ITA52 from Lipari) showed an equivocal signal in the electropherogram. Though we had repeated DNA extraction, amplification, and sequencing twice with the two problematic samples, we still received the same result after each repetition. In the alignment (with MEGA 5.1; Tamura et al. 2011), the sequences of ITA5 and ITA52 showed double peaks (Figure 1(B)), only at the variable sites that were diagnostic for each of the two parental species *P. domesticus* and *P. hispaniolensis*. This result might be indicative of heteroplasmy due to rare paternal leakage of mtDNA (Breton and Stewart 2015). The sequences from the two putative heteroplasmic individuals had therefore been discarded from further phylogeographic reconstructions by Belkacem et al. (2016).

To verify our hypothesis and to separate different haplotypes from the same sample, we designed specific primer pairs with diagnostic substitutions for each of the parental species (including one substitution at or near the 3′-end). Primer combination for the house sparrow: PassdomspecF= 5′-GAG GTA TTG CAA GGT TCA CCT C-3′, PassdomspecR= 5′-GCA ACA ATT ACA CTG CCC CCT CAC-3′; for the Spanish sparrow: PasshisspecF = GAA GTG CTG CAA GGT TCA CCC, PasshisspecR = CAC GAC AAT TAC ACT ACC CCC TCA T-3′. With these primers we amplified a 526 bp-long *ND2* fragment in a touch-down PCR with the following settings: initial degradation for 5 min at 94 °C followed by 5 cycles of 45 s at 94 °C, 45 s annealing at 57 °C and 45 s elongation at 72 °C, followed by 5 cycles 45 s at 94 °C, 45 s at 55 °C and 45 s at 72 °C, followed by 25 cycles of 45 s at 94 °C, 45 s at 53 °C and 45 s at 72 °C with a final elongation step of 5 min at 72 °C.

PCR products were purified using ExoSAP-IT™ (ThermoFischer Scientific, Waltham, MA, USA) according to the manufacturer’s instructions. Purified PCR products were sequenced on a 16-column automatic capillary sequencer (ABI 3130xl, Applied Biosystems, Foster City, CA, USA) using POP-7 as a polymer. Variable and ambiguous sites were checked visually for accuracy and validated by examining the raw data electropherogram output file using CHROMAS. Nucleotide sequences were translated into protein sequences with MEGA 5.1 in order to control for stop codons and thus to exclude nuclear mitochondrial paralogues (numts) as a potential cause of mtDNA polymorphism (Meza-Lázaro et al. 2018).

For comparison, we used ND2 sequences from 330 individuals including the data set by Belkacem et al. (2016). Sequences have been deposited at GenBank under accession numbers KX370619–KX370815 (Belkacem et al. 2016), MN488840–MN488995 (Päckert et al. 2019) and MN442613–MN442618 (sequences from heteroplasmic individuals generated for this study). Origin and metadata for all samples used for analysis can be inferred from a material table provided at Dryad under DOI: https://doi.org/10.5061/dryad.v9s4mw6qf.

The short sequences of the two putative heteroplasmic individuals inferred from separate PCRs with different primer combinations were included in the ND2 alignment and all sequences were cut down to a length of 506 bp (length of the short fragment without primer regions; 63 segregating sites). The alignment was used for the reconstruction of an mtDNA haplotype network using POPART (Leigh and Bryant 2015).

## Results and discussion

PCR with specific primer combinations yielded two strongly different ND2 haplotypes in each of the two individuals ITA5 and ITA52. Sequences inferred from independent PCR reactions with species-specific primers did not contain any double peaks and differed at those segregating sites that showed double peaks in ND2 sequences inferred from PCR with standard primers. Figure 1(B) compares a short excerpt of the chromatograms generated for bird ITA5: for the first double peak in the sequence from the left inferred with standard primes (Y = C/T) the house sparrow primer combination yielded a T and the Spanish sparrow combination a C at the same site (position 477, Figure 1(B)). The same differences between sequences inferred from PCR products with standard primers and those inferred from PCR products with specific primers are found for double peaks at sites 481, 488 and 505 (Figure 1(B)) and for all double peaks towards the 3′-end and the 5′-end of the sequences (not shown).

**Figure 2.**
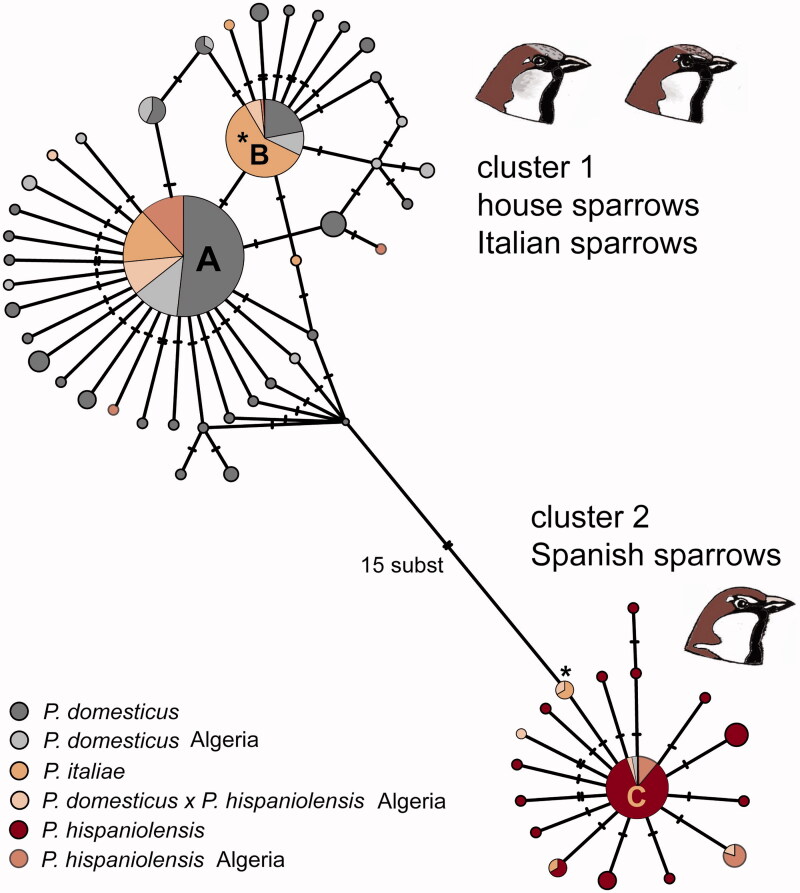
ND2 haplotype network for 330 samples of house sparrows (*P. domesticus*), Italian sparrows (*P. italiae*), and Spanish sparrows (*P. hispaniolensis*); circles represent haplotypes including information on proportions of individuals from one of the three species sharing a given haplotype (e.g. A, B, and C as the most frequent ones); numbers of substitutions indicated by dashes at connecting lines; haplotypes found in the two heteroplasmic individuals of *P. italiae* marked by asterisks.

The haplotype network clearly separated two clusters that differed by a minimum of 15 substitutions (Figure 2). The more complex cluster comprised 42 haplotypes from all Eurasian and North African house sparrow populations and several *P. italiae* populations. The most common haplotype (A) was found in 143 individuals across the entire breeding range of the house sparrow, whereas another common haplotype (B) was found in 59 individuals mainly from a circum-Mediterranean range, i.e. in Italian populations (27 out of 55 *P. italiae* carried that haplotype), North African admixed populations and Turkish house sparrow populations. The Spanish sparrow cluster comprised 17 haplotypes with the central one (haplotype C; Figure 2) found in 36 individual samples. Sequences from each of the two putative heteroplasmic individuals (ITA5 and ITA52) were indeed nested in separate clusters of the haplotype network. Sequences inferred from PCR with house sparrow primers matched haplotype B for both samples, whereas sequences inferred from PCR with Spanish sparrow primers matched one of the tip haplotypes of the *P. hispaniolensis* cluster (Figure 2). Apart from the two heteroplasmic individuals none of our *P. italiae* samples (*n* = 52) carried a haplotype from the *P. hispaniolensis* lineage. However, heteroplasmy has been documented in other populations of the stabilized hybrid *P. italiae*, e.g. from Malta (Runemark et al. 2018) and from the Italian Peninsula (Elgvin et al. 2017). It has been recently considered that heteroplasmy is a much more common phenomenon than previously anticipated (Just et al. 2015). In fact, most documented cases of mtDNA heteroplasmy originated from interspecific crosses, i.e. when mitochondrial genomes are easily distinguished, whereas cases of conspecific crosses might be easily overlooked due to greater similarity of subspecific mtDNA lineages (Mastrantonio et al. 2019). In natural populations, heteroplasmic individuals have been documented for example in *Drosophila* flies (Nunes et al. 2013; Wolff et al. 2013; Dokianakis and Ladoukakis 2014) and in sterlet sturgeons, *Acipenser ruthenus* (Dudu et al. 2012). Furthermore, paternal leakage has been documented from a tit hybrid zone (Kvist et al. 2003) and from a water frog species of hybrid origin on the Balkan Peninsula (Radojičić et al. 2015). Probably, newly emerging methods such as massive parallel sequencing will be decisive to shed light on this issue by showing a greater detection rate of heteroplasmic individuals than traditional Sanger sequencing (Just et al. 2015; Santibanez-Koref et al. 2019).
